# From Blast to Biological Uptake: Residual Dinitrotoluene as an Occupational Exposure Hazard in Explosive Ordnance Disposal

**DOI:** 10.3390/toxics14090812

**Published:** 2026-09-11

**Authors:** Gareth Collett, Kelly Johnstone, Tim Bashford, William Proud, Mia Tazi, Mieke Van Hemelrijck, Richard T. Bryan, Bryan G. Fry

**Affiliations:** 1 Faculty of Ordnance, Munitions and Explosives, University of Wales Trinity Saint David, Swansea SA1 8EW, UK; 2 Occupational Hygiene & Exposure Science Lab, School of the Environment, University of Queensland, Brisbane, QLD 4072, Australia; k.johnstone2@uq.edu.au; 3 School of Applied Computing, University of Wales Trinity Saint David, Swansea SA1 8EW, UK; tim.bashford@uwtsd.ac.uk; 4 Centre for Active Resilience and Security (CARS), Department of Physics, Imperial College London, London SW7 4AZ, UK; w.proud@imperial.ac.uk; 5 Transforming Cancer OUtcomes Through Research (TOUR), School of Cancer and Pharmaceutical Sciences, King’s College London, London SE1 9RT, UK; mia.1.tazi@kcl.ac.uk (M.T.); mieke.vanhemelrijck@kcl.ac.uk (M.V.H.); 6 Bladder Cancer Research Centre, College of Medicine and Health, University of Birmingham, Edgbaston, Birmingham B15 2TT, UK; r.t.bryan@bham.ac.uk; 7 Adaptive Biotoxicology Lab, School of the Environment, University of Queensland, Brisbane, QLD 4072, Australia

**Keywords:** dinitrotoluene, trinitrotoluene, explosive ordnance disposal, ammunition technicians, occupational exposure, demolition residues, source-pathway-receptor model, exposome

## Abstract

Repeated mixed-munition demolition may require explosive ordnance disposal personnel to re-enter a common demolition pit to inspect effects, recover debris and prepare subsequent stacks. Dinitrotoluene (DNT) is a recognised explosive ordnance occupational toxicant absorbed by inhalation, skin and ingestion. Urinary-tract malignancies and other adverse outcomes have been reported in highly exposed nitroaromatic-explosives workers. Recently, bladder-cancer incidence was documented as elevated among former British Army ammunition technicians. While these observations do not establish a causal relationship with DNT, they however provide a strong rationale for further investigation of credible exposure pathways. This study developed a scenario-based source-pathway-receptor model to examine whether residual DNT could constitute an occupational exposure source during these tasks. This model was applied to an explosive ordnance disposal operation involving 20 sequential demolitions and a documented aggregate TNT-equivalent basis of 1000 kg, used as a screening proxy because the operational inventory was predominantly TNT-filled. The model identified plausible primary inhalation from a blast-generated plume and secondary inhalation from resuspended residues, together with dermal and incidental-ingestion pathways. An important caveat is that it does not reconstruct individual dose or establish disease causation. Validation requires time-resolved post-blast and task-based personal air sampling, surface assessment, biomonitoring and detailed exposure reconstruction during representative demolition operations. As such, this study establishes crucial foundational hypotheses for future occupational hazard research into DNT exposure to explosive ordnance disposal personnel.

## 1. Introduction

Ammunition technicians and explosive ordnance disposal personnel routinely manage surplus, obsolete, recovered and unstable munitions through open demolition and other bulk-disposal procedures. These operations are primarily planned around immediate hazards such as blast, fragmentation, fire, projection and incompletely destroyed ordnance. Comparatively less attention has been directed towards the chemical residues generated by demolition and the opportunities for personnel to encounter those residues during subsequent tasks. Recent work has highlighted that explosive-ordnance activities may expose personnel to complex mixtures of energetic compounds, metals, combustion products and contaminated particulate matter [1], while an epidemiological study has reported an elevated incidence of bladder cancer among former British Army ammunition technicians [2]. The epidemiological observation does not identify a causal agent, but it strengthens the rationale for systematically characterizing previously neglected occupational exposures within this workforce.

Dinitrotoluene (DNT) comprises several nitroaromatic isomers, principally 2,4-DNT and 2,6-DNT in technical mixtures, that have been used in the manufacture of explosives, propellants and related industrial materials. DNT is a recognized explosive ordnance occupational toxicant with potential systemic uptake through inhalation, dermal absorption and incidental ingestion [3,4,5,6,7]. Historical investigations of workers involved in the manufacture, processing and disposal of nitroaromatic explosives have reported urinary-tract malignancies, renal effects and other adverse outcomes among some highly exposed groups [4,8,9,10,11,12]. The epidemiological evidence is heterogeneous and is constrained by historical exposure uncertainty, small case numbers, mixed-isomer exposure, co-exposures and other potential confounding factors. It therefore does not establish that DNT causes bladder cancer among ammunition technicians, but it provides sufficient toxicological and occupational-health concern to justify investigation of credible exposure pathways.

Biological plausibility also depends on metabolism rather than direct contact with the parent compound alone. Experimental and authoritative assessments indicate that DNT carcinogenicity and genotoxicity are metabolism-dependent: oxidative and reductive biotransformation can generate reactive intermediates and DNA-reactive products, while DNT-derived metabolites and conjugates may be eliminated in urine [3]. Urinary excretion provides a plausible route for urothelial contact, but the relevant urinary species, their concentrations and persistence, and their capacity to damage human urothelial cells have not been established. The proposed urothelial mode of action is therefore a testable biological hypothesis rather than evidence that DNT caused the reported bladder-cancer cases.

DNT is also an intermediate in the sequential nitration of toluene to trinitrotoluene (TNT) and may remain within finished TNT as a residual manufacturing impurity. The concentration and isomeric profile of residual DNT vary according to the manufacturing process, purification standard and production source [13,14,15,16,17]. Modern military-grade TNT is generally highly purified, whereas legacy, foreign-manufactured or lower-grade energetic materials may contain higher and more variable concentrations of DNT and related nitroaromatic compounds. The composition of recovered munitions encountered during operational deployments therefore cannot necessarily be inferred from contemporary specifications.

The functioning of an individual munition through its intended initiation train may produce highly efficient detonation and limited energetic residue. Bulk mixed-munition demolition is more complex. Heterogeneous stacks may contain munitions of different construction, explosive composition, confinement, geometry, age and physical condition, and they are commonly initiated externally using donor explosives rather than through the designed fuze train of each item. Field and experimental studies have shown that high-order events generally deposit comparatively small energetic residues, whereas partial and low-order reactions can leave substantially greater quantities of intact or fragmented energetic material [18,19,20,21]. Air gaps, impedance mismatch, asymmetric shock transmission, item displacement, variable donor coupling and changes in aged explosive fills provide engineering mechanisms by which high-order, partial and low-order reactions may occur within the same stack [22,23,24,25].

**Figure 1:**
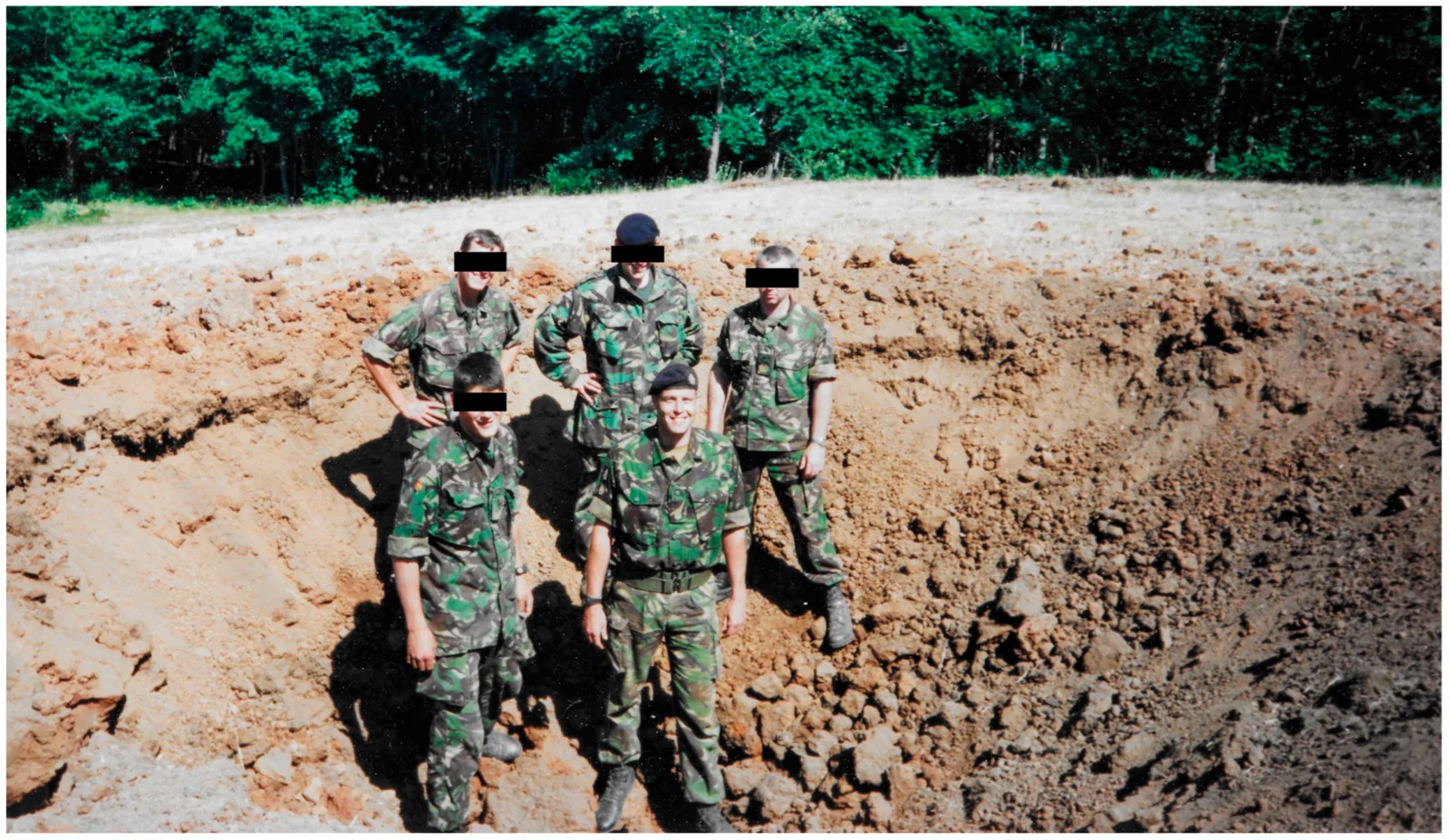
Author GC with other Operation LEATHERMAN team members (faces obscured to protect privacy) in the pit crater in Kosovo 2000.

Operational demolition differs from controlled experimental detonation because personnel may repeatedly interact with the same disturbed environment. Operation LEATHERMAN, conducted in Kosovo from 23 to 25 June 2000 with author GC taking a major leadership role, involved the recovery of approximately 60 tonnes of conventional ammunition and explosives, including a heterogeneous mixture of artillery projectiles, mortar bombs, mines, rocket-propelled grenades, propellants, demolition stores, bulk explosives and DNT-containing dynamites [26,27]. Munitions representing an aggregate TNT-equivalent basis of approximately 1000 kg required immediate disposal through 20 sequential demolition events in a single pit. The itemised operational inventory indicates that approximately 90% of this energetic equivalence was contributed by TNT-filled munitions. Although TNT equivalence and chemical TNT mass are formally distinct quantities, the predominance of TNT-filled items means that the 1000 kg value provides a reasonable screening approximation to the TNT-bearing mass involved in the operation, while remaining subject to uncertainty in individual fill masses, purity and formulation. Post-blast residues can occur as macroscopic fragments, discrete particles, condensed products, airborne particulate matter and chemicals associated with displaced soil and debris, with their distribution influenced by detonation outcome, crater dynamics, particle size, plume rise, wind, soil properties, moisture, sorption and transformation [28,29,30,31,32] (Figure 1).

Detonation may therefore create a primary airborne exposure source before residues settle or are later resuspended. Condensed explosive residues and DNT-containing fine particles can be entrained in the post-blast smoke and dust plume and transported downwind; controlled detonation experiments have identified the smoke cloud as a principal dispersal mechanism and wind direction as a major determinant of residue distribution [26]. Fine atmospheric particles can remain suspended for days to weeks and be transported over substantial distances, although concentrations in and around an open demolition pit may decline much more rapidly through dilution, advection, deposition and precipitation; atmospheric persistence should therefore not be equated with prolonged local exposure [27]. Vapour-phase 2,4-DNT and 2,6-DNT are estimated to undergo atmospheric reaction with photochemically produced hydroxyl radicals with a half-life of approximately 75 days. This is an estimated chemical degradation lifetime, not evidence that DNT vapour remains within a pit or at an occupationally relevant concentration for 75 days [28,29].

Studies at military firing and demolition ranges have demonstrated that energetic residues can persist in surface soils and that their distribution is strongly heterogeneous, particularly after inefficient or low-order events [30,31]. Analyses of military and commercial TNT have further identified DNT isomers, asymmetric TNT isomers and other nitroaromatic manufacturing by-products, supporting the need to consider the actual chemical composition of the explosive source rather than TNT mass alone [32,33]. These findings are relevant to mixed legacy munitions for which fill composition, purity and service history may be uncertain.

Dermal and secondary-transfer pathways warrant particular attention. Occupational biomonitoring during the manufacture of blasting explosives found that urinary DNT metabolites were not adequately explained by measured airborne concentrations alone, indicating that skin absorption and possibly incidental ingestion contributed to uptake [34]. Experimental work has shown that DNT can be absorbed from contaminated soil and that bioavailability is affected by soil composition, moisture, ageing and skin hydration [35]. DNT-containing energetic residues may also dissolve, migrate or be transported with sediment after deposition [36,37]. During demolition operations, contaminated soil, energetic fragments, tools, gloves, clothing and vehicles may therefore act as linked exposure media rather than as isolated sources.

Repeated entry into a demolition pit may involve inspection of demolition effectiveness, identification of incompletely destroyed items, recovery of metallic debris and preparation of subsequent stacks. Inhalation exposure may occur through two related but distinct mechanisms: direct inhalation of blast-generated particulate or vapour-phase DNT during or soon after detonation, including exposure to a drifting or recirculating plume, and secondary inhalation when deposited residues are resuspended during inspection, excavation, fragment recovery or stack preparation. These tasks also create opportunities for dermal contact with residues and incidental ingestion through contaminated hands, food, drink or tobacco products. Contamination may be transferred to vehicles, equipment, welfare areas and clean clothing unless appropriate zoning and decontamination controls are applied. The relevant occupational exposure is determined not only by total residue mass but also by the airborne fraction generated at detonation, particle size and phase partitioning, plume movement, time to re-entry, spatial distribution of deposited material, task-generated resuspension, breathing-zone concentration, duration and frequency of tasks, surface loading, skin contact, hand-to-mouth transfer and the effectiveness of controls.

Existing occupational studies demonstrate that exposure to nitroaromatic energetic compounds can occur during explosives manufacture, ammunition dismantling, demilitarisation, and remediation of ammunition-contaminated sites. Woollen et al. measured occupational DNT exposure and urinary metabolites among workers manufacturing blasting explosives, demonstrating systemic uptake and indicating an important contribution from non-inhalation pathways [34]. Letzel et al. subsequently measured airborne DNT and TNT together with urinary parent compounds and metabolites among workers mechanically disposing of military ammunition [12]. TNT exposure and biological uptake have likewise been demonstrated during ammunition-site remediation and munitions demilitarization [38,39]. However, these exposure scenarios differ fundamentally from repeated open mixed-munition demolition. We identified no published study providing time-resolved DNT measurements from the immediate post-detonation plume, personal breathing-zone measurements during subsequent demolition-pit re-entry and soil disturbance, or paired biomonitoring of personnel undertaking these EOD tasks. Consequently, directly applicable empirical exposure data are not currently available against which the present operational scenario can be calibrated. This absence of task-resolved exposure data constitutes the principal empirical gap addressed by the present source-pathway-receptor model and defines the measurements required for its subsequent field validation.

This study develops a mechanistic source-pathway-receptor model to test the hypothesis that repeated mixed-munition demolition can create a persistent DNT-containing residue reservoir that is subsequently disturbed during crater re-entry. The model is illustrated using the Operation LEATHERMAN case study and intentionally functions as a screening and hypothesis-generating analysis. It does not reconstruct the original chemical inventory, measure the proportion of DNT surviving detonation, quantify personal exposure, determine cumulative body burden or attribute disease causation. Its objectives are to integrate evidence from explosives engineering, environmental fate, occupational hygiene, toxicology and epidemiology; quantify the implications of selected scenario assumptions; and define the environmental, personal and biological measurements required to evaluate the proposed pathway under representative operational conditions.

## 2. Materials and Methods

### 2.1. Study Design and Scope

A scenario-based mechanistic model was developed to evaluate whether repeated mixed-munition demolition could create credible occupational exposure pathways for residual DNT. The analysis integrated five domains: source composition; detonation and residue-generation mechanisms; direct atmospheric release and plume transport; environmental retention, deposition and redistribution; and worker contact through inhalation, dermal absorption and incidental ingestion. The study was hypothesis-generating and did not involve prospective environmental sampling, human participants, biological specimens or individual health records.

### 2.2. Evidence Base

The model was informed by the peer-reviewed articles, government and military reports, occupational exposure resources and explosives-engineering texts cited in the manuscript. Evidence was selected for relevance to DNT toxicology and epidemiology, TNT manufacture and impurities, energetic-residue generation, mixed or low-order detonation, crater formation, environmental fate and occupational uptake. No formal systematic-review protocol, meta-analysis or quantitative evidence-grading procedure was used. Claims of novelty therefore refer to the integrated evidence base reviewed for this paper rather than to a verified exhaustive search of all published and classified literature.

### 2.3. Operational Case Study

Operation LEATHERMAN was selected as an illustrative case because it involved a documented mixed stockpile, repeated demolitions in one pit and repeated human re-entry. The operation was conducted in Kosovo from 23 to 25 June 2000. Approximately 60 tonnes of recovered conventional ammunition and explosives were managed. The TNT-filled inventory comprised of: 250 g TNT demolition blocks; Sprengstoff Cheddite No. 1 demolition charges (French stocks relabelled by the Wehrmacht); TM-46 anti-tank mines (circa 1946); 60 mm, 81 mm and 82 mm mortar rounds; 120 mm M1938 mortar rounds (circa 1939); 130 mm OF-482 and OF-482M artillery shells (circa 1954–1979); 152 mm OF-540 artillery shells; 82 mm O-881A recoilless-rifle rounds; Chinese Type 67 and Type 77 hand grenades; and Russian RGD-5 hand grenades. The Protivotankovaya Granata PG-7 anti-tank rocket-propelled grenades variants were predominantly RDX-filled.

The material requiring immediate disposal represented an aggregate TNT-equivalent basis of approximately 1000 kg [40]. Operational constraints limited each demolition stack to approximately 50 kg NEQ, resulting in 20 sequential demolitions over approximately 24 h. The 50 kg NEQ limit and the aggregate 1000 kg TNT-equivalent basis are distinct operational descriptors; no conversion between them was applied in this study. The recovered material included diverse munitions, propellants, demolition stores, bulk explosive and DNT-containing dynamites [40,41]. The itemised operational inventory indicates that approximately 90% of the aggregate energetic equivalence was contributed by TNT-filled munitions, with the principal non-TNT component being predominantly RDX-filled PG-7 variants. The operational account and contemporary photographs were used qualitatively to inform the selection of sensitivity scenarios; no image-based mass balance or quantitative retention estimate was attempted.

Three operational quantities must be distinguished when interpreting this case. The approximately 60 tonnes refers to the gross mass of recovered conventional ammunition and explosives handled during the operation; it includes munition bodies and other non-energetic material and was not used as an explosive-mass input to the model. The approximately 50 kg NEQ value describes the operational limit applied to an individual demolition stack. By contrast, the approximately 1000 kg TNT-equivalent value is the aggregate energetic basis reported for the overall disposal operation [40]. The numerical correspondence between 20 demolitions and an approximately 50 kg NEQ limit should therefore not be interpreted as the derivation 20 × 50 kg NEQ = 1000 kg TNT equivalent: NEQ is the net mass of explosive material, whereas TNT equivalence expresses energetic output relative to TNT. No NEQ-to-TNT-equivalence conversion was used in this study.

For the DNT calculations, TNT-equivalent mass is not treated as chemically identical to TNT mass. Its use as a screening basis is justified pragmatically by the itemised inventory, which indicates that approximately 90% of the aggregate energetic equivalence was contributed by TNT-filled munitions, while the principal non-TNT component comprised predominantly RDX-filled PG-7 variants. Scaling the documented 1000 kg aggregate basis by this approximately 90% contribution gives a first-order proportional comparator of approximately 900 kg. This 900 kg value is not an independent reconstruction of chemical TNT mass; it is a sensitivity comparator showing the consequence of restricting the source basis to the TNT-filled share of the reported energetic equivalence. Because the mass equations are linear in the source basis, substitution of 900 kg would reduce every modelled DNT mass by 10% (for example, the potential post-reaction range would become 4.50 to 180 g rather than 5.00 to 200 g). Conversely, the 1000 kg basis produces values 11.1% higher than this comparator. This difference is modest relative to the deliberately broad uncertainty ranges applied to residual energetic-material fraction, DNT fraction, and local retention, and it does not alter the source-pathway-receptor interpretation. The 1000 kg basis was therefore retained to preserve the documented operational scale while remaining explicitly identified as a screening approximation rather than an analytical TNT inventory.

### 2.4. Scenario Construction

The primary calculations retained the documented 1000 kg TNT-equivalent operational basis for consistency with the case record, with its provenance and interpretation detailed in Section 2.3. The separately reported approximately 50 kg NEQ stack limit was not used to derive this aggregate value, and no NEQ-to-TNT-equivalence conversion was applied. As a source-basis sensitivity check, a 900 kg proportional comparator was considered from the approximately 90% contribution of TNT-filled munitions. Because all mass calculations are linear in the source basis, this comparator scales every DNT output to 90% of the values reported for the 1000 kg basis; it is not treated as a reconstructed chemical TNT inventory. Three residual energetic-material fractions were applied: 0.5%, 1.0% and 2.0%. The upper value was limited to 2.0% to reflect operational observations that most munitions functioned effectively and only a small proportion visibly deflagrated, while still allowing for incomplete reactions within some events. Three DNT fractions were then applied to the residual mass: 0.1%, 0.5% and 1.0%, producing nine potential post-reaction DNT scenarios before local retention. A sensitivity analysis then applied illustrative local-retention fractions of 50%, 75% and 90%. These values were selected to test the persistence of the proposed accumulation pathway across markedly different assumptions and were not treated as measured fallback rates or as lower, central and upper estimates of the true retained fraction. The local environment was defined as the crater floor, walls, rim and immediately adjacent disturbed soil. Because the mass basis is an operational approximation rather than an analytical assay, and none of the retention fractions was measured specifically for DNT, all outputs were interpreted as conditional screening values rather than reconstructed environmental masses.

### 2.5. Crater-Volume Calculation

The final crater was represented as a semi-ellipsoid using the measured dimensions of 8.0 m length, 7.0 m width and 1.9 m depth. The semi-major axis a was 4.0 m, the semi-minor axis b was 3.5 m and the vertical semi-axis c was 1.9 m. Crater volume was calculated as:

(1) $$V\;=\;\frac{2}{3}\pi abc\;=\;\frac{2}{3}\pi \;\times \;4.0\;m\;\times \;3.5\;m\;\times \;1.9\;m\;=\;55.7\;m^{3}$$

The calculated volume was used only as a geometric comparator. The crater was not treated as a sealed chamber, and no assumption was made that airborne contaminants would remain uniformly suspended for a specified duration.

### 2.6. Potential and Conditionally Retained DNT Calculations

For each scenario, the residual energetic-material mass was calculated from the selected mass basis and residual fraction:

(2) $$M_{\mathrm{residual}}\;=\;M_{\mathrm{basis}}\;\times \;f_{\mathrm{residual}}$$

Potential post-reaction DNT mass before application of any crater-retention factor was calculated as:

(3) $$M_{\mathrm{DNT},\mathrm{potential}}\;=\;M_{\mathrm{residual}}\;\times \;f_{\mathrm{DNT}}$$

Conditional retained DNT mass was then calculated as:

(4) $$M_{\mathrm{DNT},\mathrm{retained}}\;=\;M_{\mathrm{DNT},\mathrm{potential}}\;\times \;f_{\mathrm{retention}}$$

where $M_{\mathrm{basis}}$ was 1000 kg on a TNT-equivalent basis; $f_{\mathrm{residual}}$ was 0.005, 0.01 or 0.02; $f_{\mathrm{DNT}}$ was 0.001, 0.005 or 0.01; and $f_{\mathrm{retention}}$ was 0.50, 0.75 or 0.90. These values were treated as local-retention sensitivity points rather than measured fallback or deposition fractions. Calculations were performed directly from these equations and rounded to three significant figures for presentation.

### 2.7. Dimensional Benchmark Illustrations

A limited dimensional scale illustration was calculated using the ACGIH value of 0.2 mg/m^3^ and the 55.7 m^3^ crater volume:

(5) $$M_{\mathrm{benchmark}}\;=\;C_{\mathrm{benchmark}}V\;=\;0.2\;\frac{\mathrm{mg}}{m^{3}}\;\times \;55.7\;m^{3}\;=\;11.1\;\mathrm{mg}$$

This calculation was included solely to illustrate dimensional scaling and was not expressed as a proportion of the potential post-reaction or conditionally retained DNT masses. It does not represent contaminant generation, airborne suspension, atmospheric dispersion or personal exposure. No mobilisation threshold, time-weighted average, breathing-zone concentration, particle-size distribution, respiratory uptake or occupational-limit compliance was calculated.

For the mean-equivalent soil-concentration illustration, the apparent crater volume was multiplied by an assumed soil bulk density of 1350 kg/m^3^, yielding an equivalent displaced-soil mass of approximately 75,200 kg. Conditional local DNT masses were divided by this equivalent mass to express a uniform-concentration scale in mg/kg. The calculation was not treated as an estimate of the actual soil mass, spatial concentration distribution, bioavailable fraction or occupational risk, and no Regional Screening Level exceedance was inferred.

### 2.8. Model Assumptions and Limitations

The model therefore evaluates plausibility rather than probability or risk, and it should be updated as measured source, environmental and personal-exposure data become available (Table 1).

## 3. Results

The outputs of the screening model are presented below. The underlying equations, scenario construction and limitations are detailed in the Section 2.

### 3.1. Operational Scenario and Source Assumptions

The model used the documented operational pattern of Operation LEATHERMAN (Table 2), in which munitions representing an aggregate TNT-equivalent basis of approximately 1000 kg were disposed of through 20 sequential mixed-munition demolitions, each subject to an operational limit of approximately 50 kg net explosive quantity (NEQ), within a single demolition pit over approximately 24 h [40]. As detailed in Section 2.3, the 1000 kg value was not obtained by multiplying 20 demolitions by the approximately 50 kg NEQ stack limit; NEQ and TNT equivalence are distinct descriptors, and no conversion between them was applied. The itemised inventory indicates that approximately 90% of the aggregate energetic equivalence was contributed by TNT-filled munitions, while the principal non-TNT component comprised predominantly RDX-filled PG-7 variants. This approximately 90% contribution gives a 900 kg first-order proportional comparator, under which every DNT mass output is 10% lower than under the 1000 kg basis. The comparator is not an analytical reconstruction of chemical TNT mass. The documented 1000 kg basis was retained as a mildly conservative screening approximation because exact fill masses, purity, and DNT content were not measured.

### 3.2. Engineering Basis for Residue Generation

The heterogeneous stack configuration creates several mechanisms that can reduce the probability that every item undergoes efficient high-order detonation. Air gaps and discontinuous contact alter shock coupling between the donor charge and individual munitions. Differences in casing geometry, explosive fill, confinement and orientation produce impedance mismatch and asymmetric loading. Items at the periphery of the stack may be displaced or kicked out before complete reaction. Ageing, exudation, phase separation, internal voids and changes in crystalline structure can further impair uniform shock transmission through legacy fills. These mechanisms support the plausibility of a mixed outcome in which high-order, partial and low-order reactions occur during the same demolition event [18,19,20,21,22,23,24].

Residue generation and local retention are not the only relevant post-detonation processes. Surviving DNT or DNT-containing condensed material may be emitted directly into the primary smoke and dust plume as fine particulate matter, while a fraction may occur in the vapour phase. Particle size, thermal history, plume buoyancy, wind speed and direction, atmospheric stability, pit geometry and time to re-entry will govern whether this material is inhaled near the demolition point, transported downwind or deposited for later contact. The present mass model does not quantify this immediate airborne branch or the partitioning of DNT between vapour, particle-bound and deposited phases.

Operational observations provide a qualitative basis for treating local retention as plausible but uncertain. Contemporary photographs show little obvious accumulation of coarse ejecta beyond the crater margins, although photographs cannot resolve the transport of fine soil or contaminated particulate matter. Repeated use of the established pit would also have caused successive demolitions to rework material already present within the crater and its immediate margins. The donor charges were positioned above the munition stacks, plausibly increasing coupling of explosive energy into the underlying pit material. Operational accounts indicate that most munitions functioned effectively and that only a small proportion visibly deflagrated. Together, these observations support the use of a limited residual-material range and the plausibility of substantial local incorporation within the crater floor, walls, rim and immediately adjacent disturbed soil, but they do not establish a specific retention percentage [40].

### 3.3. Crater Geometry

**Figure 2:**
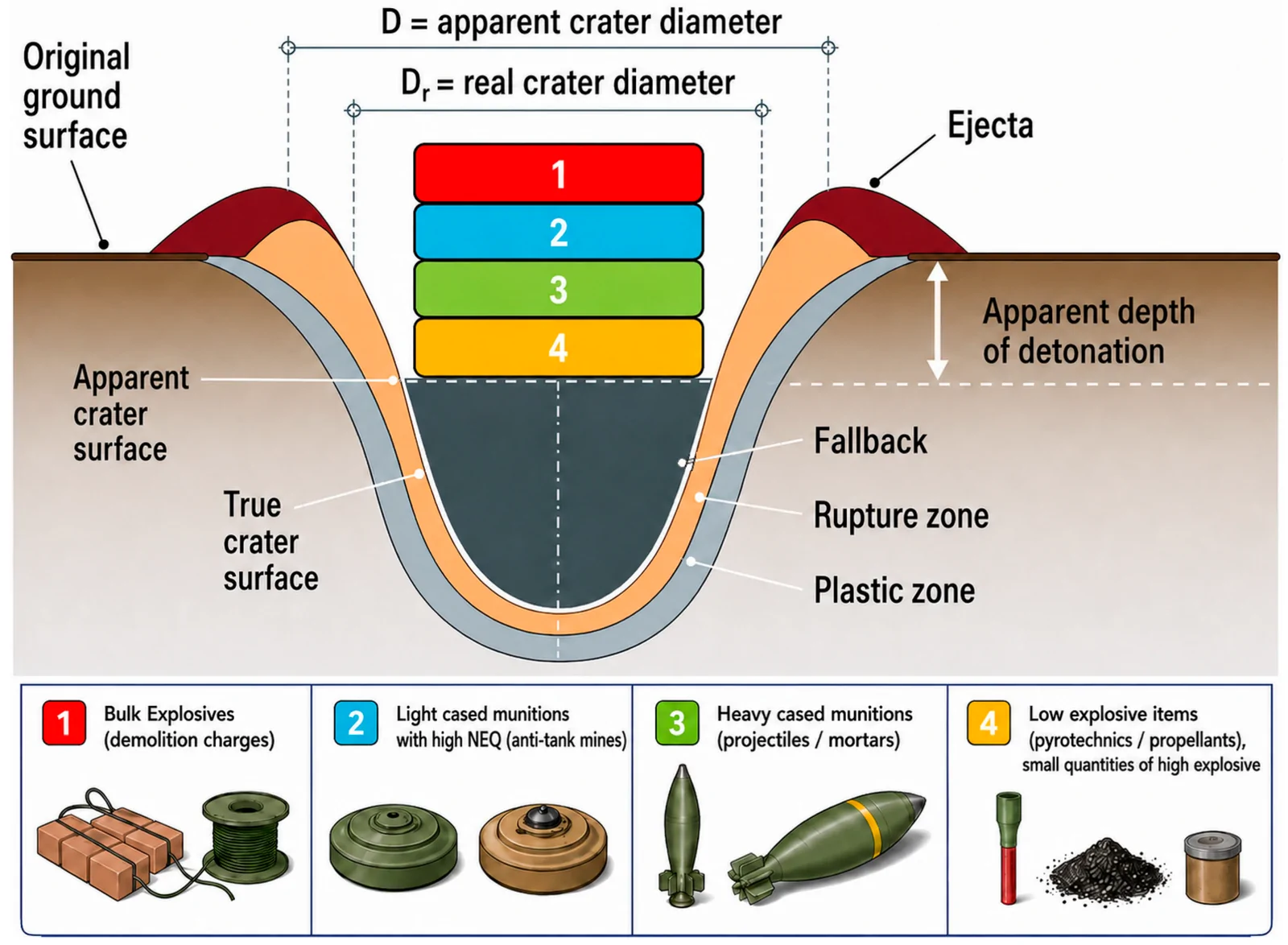
Representative schematic of a heterogeneous demolition stack showing crater-formation geometry and detonation-depth classification for mixed energetic materials.

The measured final crater dimensions of 8.0 m × 7.0 m × 1.9 m were represented as a semi-ellipsoid with semi-axes of 4.0 m and 3.5 m and a depth of 1.9 m. The principal crater features and the distinction between apparent and true crater geometry are illustrated in Figure 2. The figure also shows the conceptual positioning of different categories of energetic material relative to the apparent depth of detonation. The resulting calculated crater volume was 55.7 m^3^. This volume was used as a geometric descriptor and, in Section 2.5, only for a limited dimensional scale illustration; it does not represent a closed or uniformly mixed occupational atmosphere.

### 3.4. Scenario-Estimated Potential and Conditionally Retained DNT Mass

Across the nine combinations of residual-material fraction and DNT fraction, the potential post-reaction DNT mass before application of any local-retention factor ranged from 5.00 g to 200 g (Table 3). A sensitivity analysis was then performed using illustrative local-retention fractions of 50%, 75% and 90%. Conditional locally retained masses ranged from 2.50 to 100 g at 50% retention, 3.75 to 150 g at 75% retention and 4.50 to 180 g at 90% retention. These values are conditional screening outputs rather than measured environmental concentrations and do not establish how much DNT was present in the original stockpile, survived detonation, was retained within the local working environment or remained bioavailable after deposition. The sensitivity analysis illustrates how the selected source-composition and local-retention assumptions influence the conditional local DNT burden. It does not establish that the assumed retention fractions occurred, that retained DNT was bioavailable or that occupational exposure resulted.

The 50% scenario allows half of the estimated residual DNT to have been transported beyond the local crater environment, whereas the 75% and 90% scenarios represent progressively greater incorporation within the crater floor, walls, rim and immediately adjacent disturbed soil. The sensitivity points do not quantify the actual retained fraction. Rather, they test whether the proposed local accumulation pathway remains plausible across markedly different assumptions, including a scenario in which half of the estimated residual DNT is dispersed beyond the principal working area.

### 3.5. Dimensional Benchmark Comparisons and Their Interpretive Limits

International occupational exposure limits for DNT vary substantially. Current eight-hour time-weighted-average values range from approximately 0.15 mg/m^3^ to 1.5 mg/m^3^ [42]. Most limits apply to mixed DNT isomers and carry a skin notation. From 1 December 2026, Australia will no longer assign DNT a numerical workplace exposure limit, instead regulating it as a non-threshold genotoxic carcinogen for which elimination, substitution and minimisation of exposure are required [43]. The ACGIH threshold limit value–time-weighted average (TLV–TWA) for DNT is 0.2 mg/m^3^ with a skin notation [5]. Multiplying this concentration by the calculated 55.7 m^3^ apparent crater volume yields 11.1 mg. Relative to the lowest conditionally retained DNT mass in the screening model (2.50 g), 11.1 mg is equivalent to 0.45%. This is a dimensional mass–volume comparison only. The crater is an open and dynamically ventilated environment, and the TLV–TWA is a personal breathing-zone concentration averaged over a work shift; the calculation therefore cannot be interpreted as a mobilisation threshold, a predicted airborne concentration or evidence that the TLV would be reached or exceeded.

Both the primary blast-generated plume and secondary task-generated dust clouds may produce breathing-zone concentrations that differ substantially from a crater-wide volumetric average, but neither the direction nor magnitude of those differences can be quantified without measurement. Fine particles may remain suspended and travel downwind after the visible plume has dissipated, whereas coarse material deposits more rapidly; local persistence depends on particle size, meteorology, terrain and the interval before re-entry. The estimated 75-day atmospheric half-life of vapour-phase DNT describes chemical degradation by hydroxyl-radical reaction and must not be used as a surrogate for local plume residence time or worker exposure duration. A valid occupational comparison requires time-resolved air monitoring beginning with the post-blast plume and continuing through re-entry and soil-disturbance tasks, together with personal breathing-zone sampling, appropriate inhalable and respirable fractions, chemical analysis of DNT in collected particulate matter, vapour-phase sampling where analytically feasible, exposure duration, meteorological context and documentation of respiratory protection. Dermal and incidental-ingestion routes require separate assessment because an airborne TLV does not represent total systemic uptake.

A second dimensional calculation was used to examine the scale of the modelled local burden if it were expressed as a mean-equivalent soil concentration. Applying an illustrative bulk density of 1350 kg/m^3^ to the 55.7 m^3^ crater volume gives an equivalent displaced-soil mass of approximately 75,200 kg. Across the scenarios using a 2.0% residual energetic-material fraction, conditionally retained DNT ranged from 10.0 to 180 g when all modelled DNT fractions and local-retention assumptions were considered. Uniform distribution through the equivalent soil mass would correspond to approximately 0.13–2.39 mg/kg. The narrower 100–180 g range represents only the highest applied DNT fraction and therefore should not be presented as the full range of the 2.0% residual-material scenarios.

This mean-equivalent concentration is not a measured soil concentration. The apparent crater volume is a geometric void rather than a defined mass of retained soil, the 1350 kg/m^3^ bulk density was not measured at the site, and residues would not be expected to distribute homogeneously. Repeated pit reworking, soil fallback, fragment retention and particle transport could produce pronounced lateral and vertical heterogeneity, with local concentrations either above or below the calculated mean. Photographs and operational descriptions support the possibility of local incorporation but cannot resolve fine-particle transport or identify the locations of DNT hotspots.

No Regional Screening Level exceedance ratio was calculated. Generic U.S. EPA Regional Screening Levels are scenario-specific risk-based screening values rather than occupational exposure limits or cleanup standards [44]. A residential-soil value such as 0.8 mg/kg cannot be applied without confirming the current table, target risk and hazard quotient, analyte definition, land-use scenario and relevance to demolition work. Any soil-based risk interpretation would therefore require current scenario-appropriate screening values, measured DNT isomer concentrations, site-specific soil properties, spatial sampling and an exposure model relevant to demolition personnel.

Low-order reactions or deflagrations could leave greater residue masses than efficient high-order detonations, but the available operational account indicates that such outcomes were uncommon during Operation LEATHERMAN. The present calculations therefore retain the 2.0% residual energetic-material fraction as the upper screening scenario and do not use the benchmark comparisons to expand the modelled residue range.

### 3.6. Source-Pathway-Receptor Interpretation

**Figure 3:**
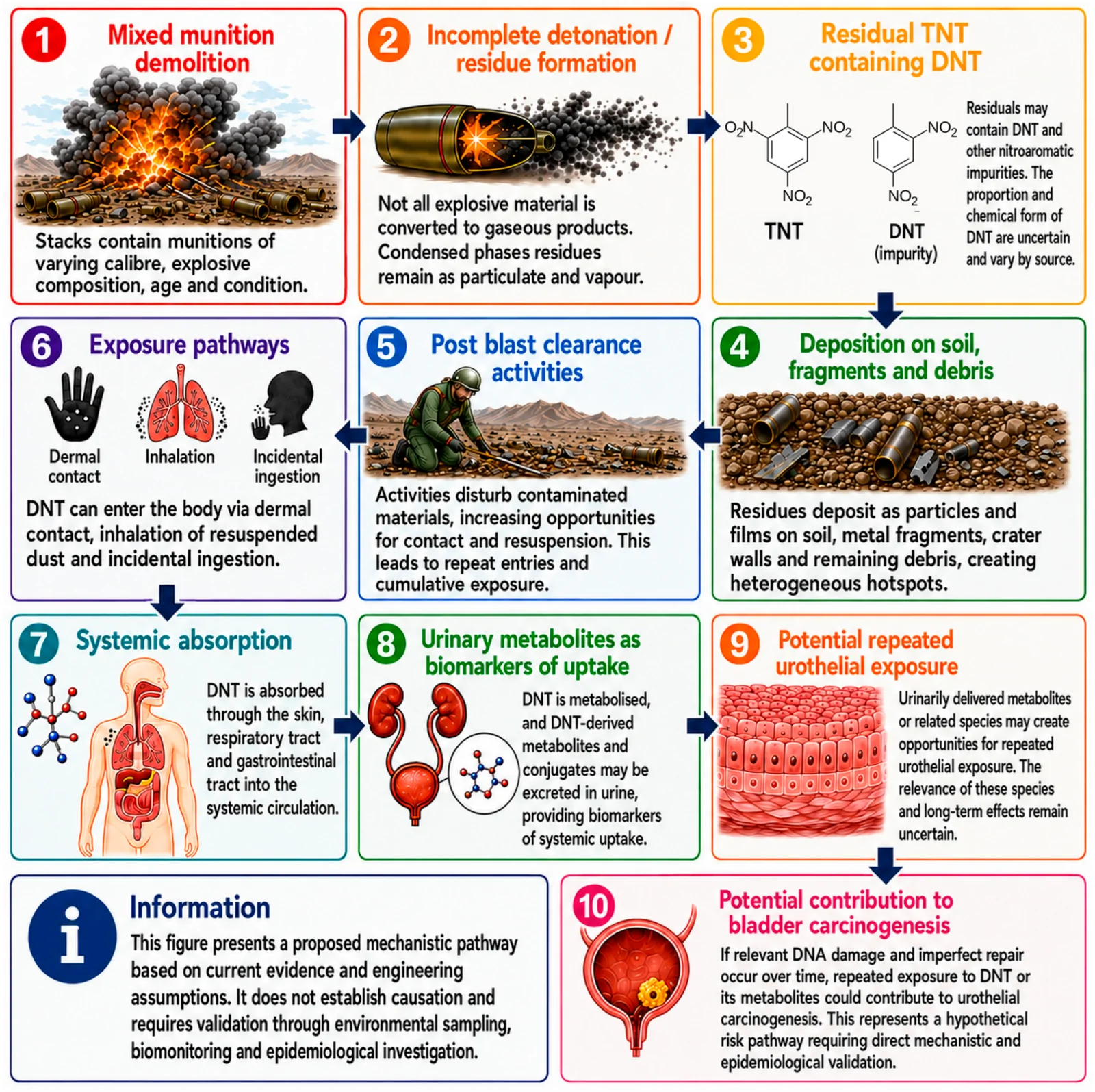
Proposed mechanistic pathway linking mixed-munition demolition residues to potential DNT exposure and urothelial effects. Incomplete or partial detonation may generate an immediate airborne plume containing DNT-bearing particulate matter and potentially vapour-phase DNT, while also leaving residual energetic material containing DNT and related nitroaromatic manufacturing impurities. Airborne material may be inhaled directly before deposition or transported downwind. Deposited residues may occur heterogeneously on soil, fragments and debris and be subsequently disturbed during post-blast clearance, creating a secondary inhalation pathway together with opportunities for dermal contact and incidental ingestion. Following systemic absorption, DNT is metabolised and urinary metabolites may provide biomarkers of uptake and a potential route for urothelial contact. Repeated exposure is hypothesised to contribute to cellular stress or genotoxic effects and, potentially, to bladder carcinogenesis.

For occupational exposure to occur, DNT must first be present within the original energetic-material inventory and survive the demolition process in a chemically identifiable form. From that point, the pathway can divide into primary and secondary airborne branches. In the primary branch, blast-generated smoke, dust and condensed particles may carry DNT directly into the air at detonation; vapour-phase DNT may also be present. Personnel could inhale this material during plume drift, incomplete atmospheric clearance or early re-entry, and downwind personnel may be exposed even without entering the crater. In the secondary branch, surviving DNT is deposited within soil, fragments, debris or contaminated surfaces and is later resuspended during inspection, excavation, fragment recovery, vehicle movement or preparation of subsequent demolitions. Deposited material also provides sources for dermal transfer and incidental ingestion. Fine particles can have longer atmospheric residence times than coarse ejecta, but the persistence of an occupationally relevant concentration in or around the pit cannot be inferred from general aerosol lifetimes or from the estimated 75-day atmospheric degradation half-life of vapour-phase DNT. Particle size, plume rise, wind, turbulence, precipitation, terrain, pit recirculation and the delay before re-entry must be measured or reconstructed. Following absorption by any route, DNT is metabolised, and DNT-derived metabolites and conjugates may be excreted in urine, creating a biologically plausible but unconfirmed route through which repeated exposure could contribute to urothelial contact with DNT-derived species. The proposed sequence from mixed-munition demolition through immediate airborne release and/or environmental deposition, occupational exposure, systemic uptake, urinary excretion and potential urothelial effects is summarised in Figure 3. This pathway is mechanistic and hypothesis-generating and does not establish individual dose or disease causation. The proposed cellular mechanism underlying the urothelial component of the pathway is considered in Section 3.8.

**Figure 4:**
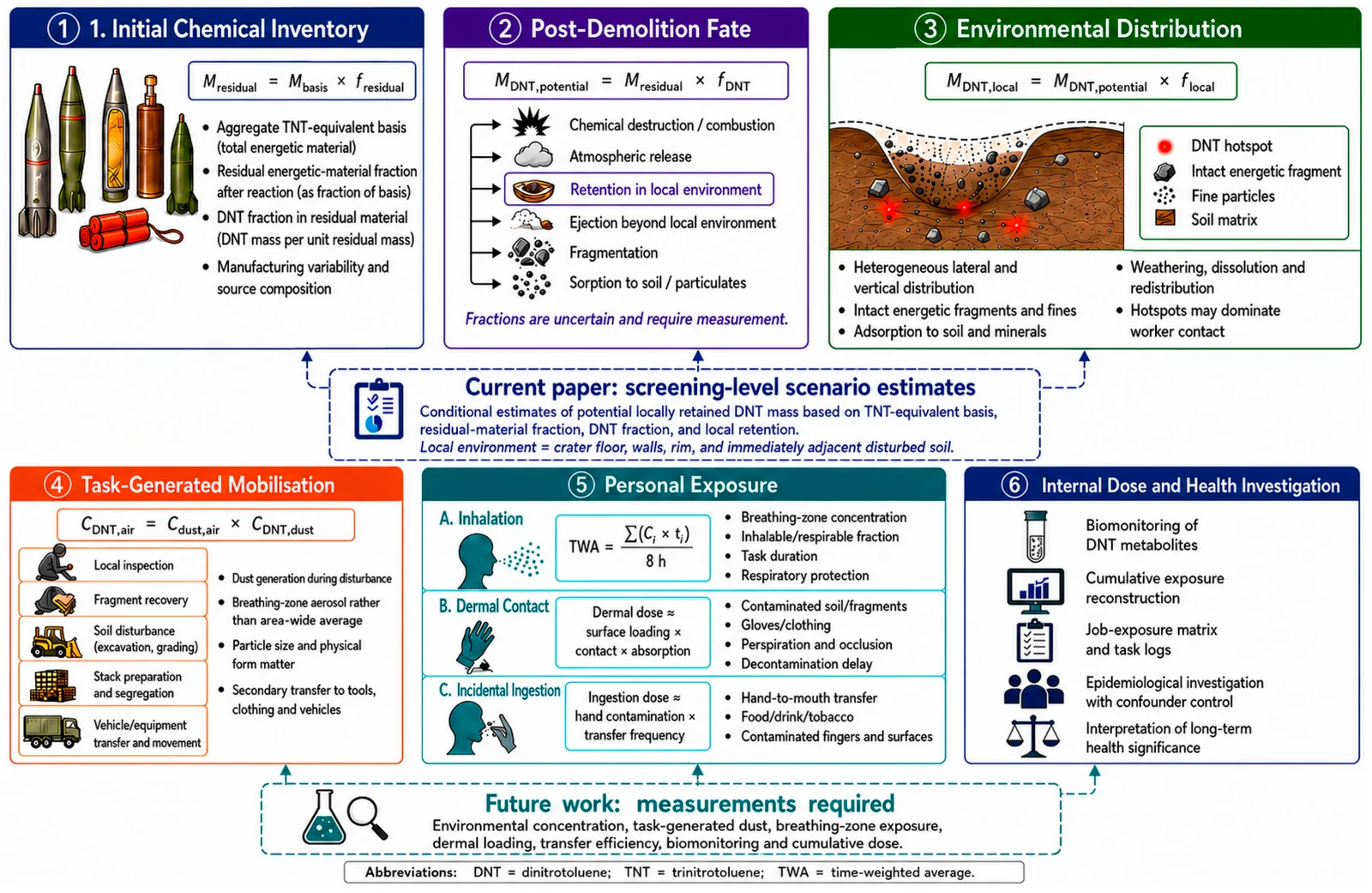
Quantitative framework linking initial DNT inventory, post-demolition fate, environmental distribution, task-generated mobilisation and personal exposure with downstream internal-dose assessment and health investigation. The current study provides screening-level estimates of potential post-reaction DNT mass and conditional retained mass using assumed inventory and residual-material fractions together with illustrative retention fractions. Quantitative occupational exposure assessment additionally requires measurement of DNT survival and phase partitioning, direct blast-generated particulate and vapour-phase emissions, plume movement, spatial deposition, soil and dust concentrations, secondary task-generated aerosol, breathing-zone exposure, dermal loading, transfer efficiency, exposure duration and biological uptake. Retained contaminant mass alone cannot be interpreted as personal exposure or compliance with an occupational exposure limit.

Progression from a potential DNT source to measurable occupational exposure requires several sequential and independently uncertain processes. These include determination of the initial DNT inventory; survival, phase partitioning and direct atmospheric release during demolition; plume transport and deposition; environmental distribution within soil and debris; secondary mobilization during specific tasks; transfer to the breathing zone or skin; and subsequent uptake. The quantitative relationships and principal measurement requirements linking these stages are summarised in Figure 4.

The present analysis addresses only the early stages of this framework by generating screening-level estimates of potential post-reaction DNT mass and evaluating conditional retention across illustrative sensitivity scenarios. It does not estimate the mass or concentration of DNT released directly into the post-blast plume. Quantitative assessment of occupational exposure would additionally require time-resolved plume measurements, vapour and particle-phase characterisation, environmental concentration data, task-specific dust measurements, personal breathing-zone sampling, surface and dermal loading measurements, exposure-duration records, transfer-efficiency estimates and biological monitoring.

### 3.7. Occupational Exposure Routes

#### 3.7.1. Inhalation

Inhalation exposure may begin with the demolition event itself rather than only with later soil disturbance. Detonation can generate a primary smoke and dust plume containing fine condensed particles, incompletely reacted energetic material and potentially vapour-phase DNT. Depending on wind and atmospheric conditions, this plume may drift across personnel positions, move downwind or recirculate locally before complete dilution and deposition. Fine particles may remain airborne after the visible plume has dispersed, although this does not demonstrate that elevated concentrations persist in the pit for days or weeks. A second inhalation source arises when inspection, excavation, fragment recovery and preparation of subsequent stacks resuspend DNT-bearing soil and debris within the operator’s breathing zone [1]. Exposure from both sources will depend on DNT survival and phase partitioning, particle-size distribution, airborne concentration, plume trajectory, time to re-entry, local wind and atmospheric mixing, task duration and respiratory protection. Area or crater-wide averages cannot substitute for time-resolved personal sampling because both the initial plume and later task-generated clouds may be spatially heterogeneous and short-lived.

#### 3.7.2. Dermal Contact

DNT carries a skin notation under the cited occupational frameworks [5,6,7]. Personnel may contact contaminated soil, energetic fragments, metallic debris, tools, demolition stores, gloves, clothing and personal protective equipment [1]. Perspiration, prolonged occlusion, damaged skin, repeated handling and delayed decontamination may increase the opportunity for transfer and absorption [1]. Contaminated gloves or clothing may also prolong contact after personnel leave the crater [34,35].

#### 3.7.3. Incidental Ingestion and Secondary Transfer

Hand-to-mouth transfer can occur when contaminated hands, gloves or equipment contact food, drink, tobacco products or the face [1]. Residues may also be transferred to vehicles, accommodation, welfare areas and clean equipment [1]. Although difficult to quantify retrospectively, repeated low-level transfer may contribute to cumulative uptake and should be included in task-based exposure assessment rather than treated as an ancillary pathway.

### 3.8. Proposed Urothelial Mode of Action for Dinitrotoluene

Dinitrotoluene is more appropriately regarded as a metabolically activated nitroaromatic carcinogen candidate than as a direct-acting urothelial carcinogen. Its disposition, biotransformation and genotoxicity vary with isomer, tissue, species and exposure conditions. DNT-specific studies support hepatic oxidation of the methyl substituent to dinitrobenzyl alcohol and aldehyde derivatives, conjugation—particularly glucuronidation—and biliary excretion. Intestinal deconjugation and microbial nitroreduction can generate reduced metabolites that are reabsorbed, allowing enterohepatic cycling and further hepatic processing; the relative contribution of these pathways and the resulting metabolite profiles differ between 2,4-DNT and 2,6-DNT [3,44,45,46,47,48,49,50]. More broadly, nitroreduction of nitroaromatic compounds can form nitroso and hydroxylamine intermediates capable of redox cycling and electrophilic reactions, providing plausible routes to reactive oxygen species, oxidative stress, covalent macromolecular binding and DNA-adduct formation [51].

The DNT-specific genotoxicity evidence is derived primarily from bacterial assays, rodent liver models and whole-animal studies rather than from human urothelial cells. Both 2,4-DNT and 2,6-DNT have formed hepatic DNA adducts in rats, with quantitatively greater adduct formation generally reported for 2,6-DNT, and 2,6-DNT has produced positive findings in bacterial mutagenicity and rat-liver comet assays [52,53]. If oxidative or electrophilic lesions are not repaired, they could be fixed as mutations affecting genome maintenance, cell-cycle control or apoptosis. Experimental carcinogenicity studies and authoritative evaluations therefore support DNT as a metabolically activated animal carcinogen, but do not establish the urinary bladder as a direct target tissue or demonstrate a bladder-specific mechanism in humans [3,29,54].

A urothelial mode of action would require DNT-derived metabolites or conjugates to enter the systemic circulation, be delivered into urine, remain sufficiently stable and bioavailable, and contact or enter urothelial cells. Nitrotoluene-derived haemoglobin adducts and urinary metabolites have been detected in occupationally exposed workers, confirming systemic uptake and metabolic processing [55], but these findings do not identify the urinary species responsible for any urothelial effect. Direct measurements of DNT-derived DNA adducts, oxidative lesions or mutations in human urothelial tissue are lacking. The proposed sequence is therefore: metabolic activation; urinary delivery of parent compound, metabolites or conjugates; urothelial uptake or luminal deconjugation; oxidative or electrophilic DNA damage; incomplete repair; and fixation of mutations during compensatory cell proliferation. Repeated exposure could increase the cumulative probability of these events. Cycles of epithelial injury, inflammation and compensatory repair are biologically plausible, but have not been demonstrated for DNT in human urothelium; the identity, concentration and persistence of the relevant urinary species remain unknown.

This sequence is analogous, but not equivalent, to the established mechanism of aromatic-amine bladder carcinogenesis, in which hepatic activation is followed by urinary transport of reactive metabolites or conjugates, luminal release, urothelial DNA-adduct formation, oxidative injury and proliferative responses [10,56,57]. The analogy supports biological plausibility but cannot substitute for DNT-specific evidence because DNT is a nitroaromatic compound with distinct activation pathways and metabolite profiles. Direct testing should use primary human urothelial cells or organoid models exposed to chemically defined DNT metabolites and, where feasible, biologically relevant urinary metabolite mixtures. Priority endpoints include intracellular reactive oxygen species; 8-oxo-7,8-dihydro-2′-deoxyguanosine (8-oxodG); γ-H2AX; conventional and formamidopyrimidine DNA glycosylase (Fpg)-modified comet assays, with the latter improving sensitivity to oxidised purines; covalent DNA adducts; mutation spectra; cytotoxicity; inflammatory signalling; and compensatory proliferation. These mechanistic studies should be integrated with occupational biomonitoring and exposure reconstruction [58]. The available worker reports establish exposure opportunity and health concern, but not this molecular sequence or DNT causation [4,11,12]. However, evidence from studies in firefighters (who experience exposure to toluene amongst other compounds in smoke) suggests changes in miRNA expression and DNA methylation lead to alterations in the epigenetic regulation of gene promoters [58]. Although not directly linked to toluene exposure, DNA methylation changes are considered to be early events in urothelial carcinogenesis [59,60,61,62].

### 3.9. Cumulative Exposure and Occupational Significance

The defining feature of the proposed pathway is repetition. During one operation, personnel may enter the same pit after multiple demolitions; across a career, they may perform similar tasks at many sites containing different explosive formulations and degrees of contamination. The total occupational burden may therefore reflect repeated episodes involving multiple energetic chemicals and metals rather than a single DNT exposure. The reported bladder-cancer incidence among former ammunition technicians [2] provides a rationale for investigation but cannot be used to identify DNT as the causal agent. DNT should be evaluated as one component of a broader demolition-related exposome.

### 3.10. Research and Operational Priorities

The model identifies several immediate priorities for validation and exposure control:

- Environmental sampling of the immediate post-blast plume, deposited particulate matter, crater soil, visible energetic fragments, settled dust and adjacent surfaces for 2,4-DNT, 2,6-DNT, TNT and related nitroaromatic compounds.
- Time-resolved area and personal air sampling beginning as soon as operationally safe after detonation and continuing through crater re-entry and soil-disturbance tasks, using inhalable and respirable fractions, real-time particulate monitoring and, where feasible, vapour-phase DNT sampling.
- Surface-wipe, glove-wipe and equipment-wipe sampling to characterise dermal loading and transfer between dirty and clean zones.
- Pre- and post-task biological monitoring using analytically appropriate DNT metabolites, with sampling times selected according to toxicokinetics and operational feasibility.
- Detailed task logging covering munition type, explosive formulation where known, demolition outcome, plume direction and duration, delay to re-entry, number and duration of crater entries, soil disturbance, weather, respiratory protection, glove use and decontamination.
- Development of a retrospective job-exposure matrix for epidemiological analysis, with appropriate control for smoking, age, latency, deployment history and co-exposures.
- Review demolition procedures to minimize dust generation and worker exposure, including the use of remote inspection technologies, wet methods (where safe and practicable), dirty–clean zoning, decontamination points, improved management of contaminated clothing, vehicles and accommodation facilities, and optimization of respiratory protection where residual exposure cannot be otherwise controlled.

**Figure 5:**
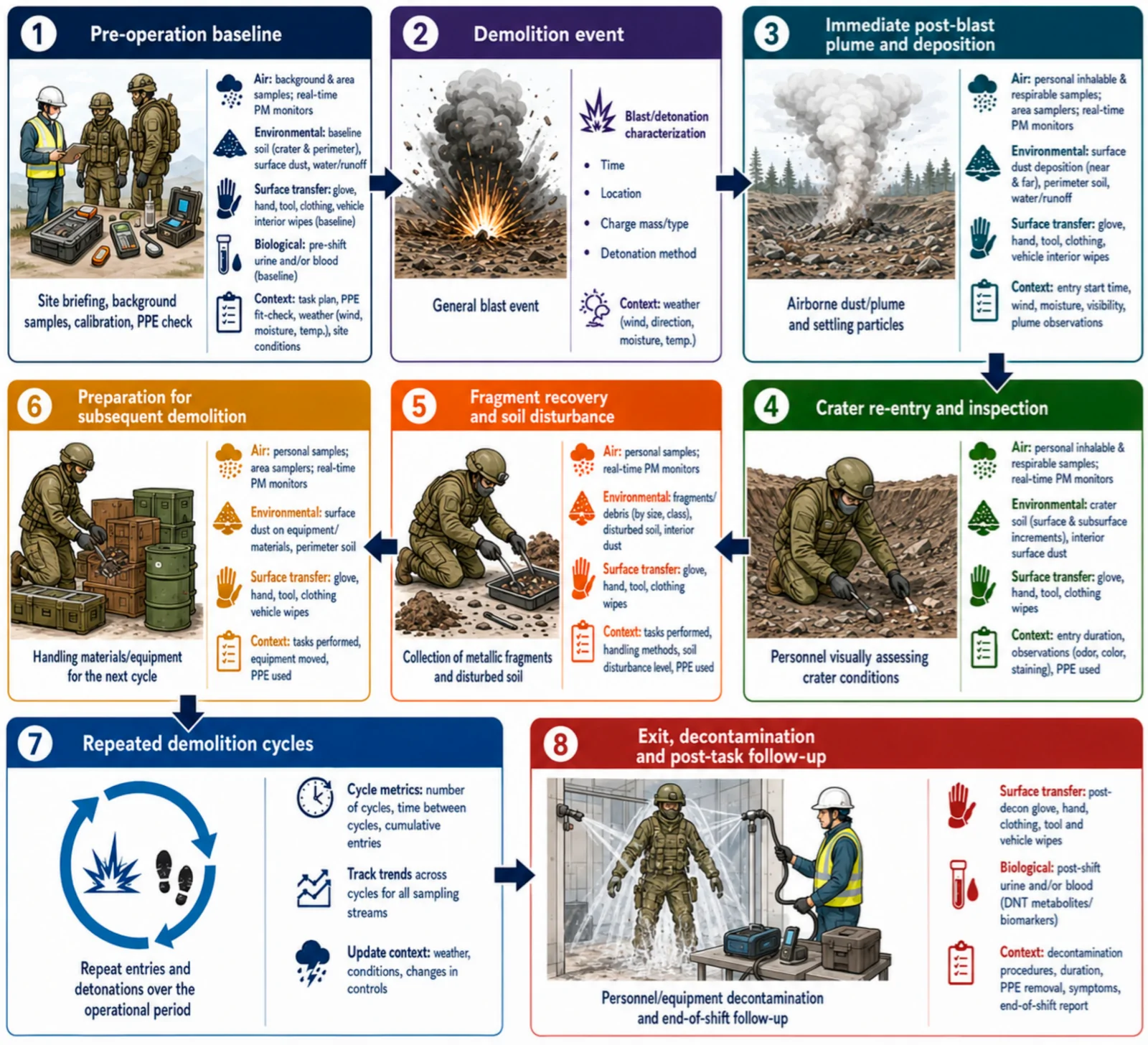
Proposed field-validation design for repeated mixed-munition demolition operations. Environmental, personal-exposure, surface-transfer, biological and contextual measurements are aligned with eight successive operational stages: pre-operation baseline assessment; the demolition event; immediate post-blast plume and deposition; crater re-entry and inspection; fragment recovery and soil disturbance; preparation for the subsequent demolition; repeated demolition cycles; and exit, decontamination and post-task follow-up. The design is intended to distinguish exposure associated with the post-blast plume, crater entry, task-generated dust, direct handling, dermal transfer and secondary contamination, while tracking changes across repeated cycles. It is a conceptual sampling framework and does not reconstruct a specific operation, quantify individual exposure or estimate personal dose.

**Figure 6:**
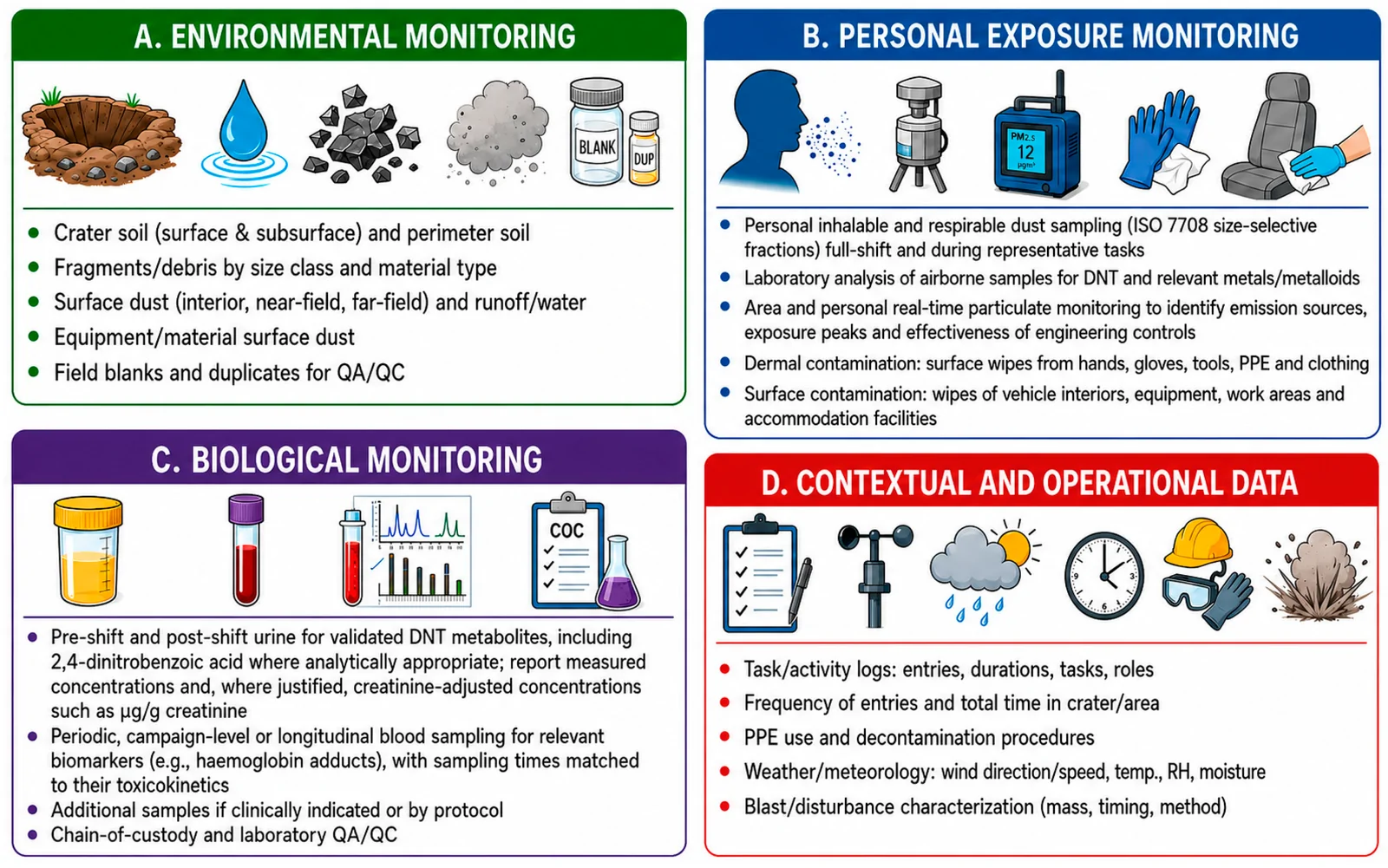
Proposed sampling streams for field validation of occupational exposure during repeated mixed-munition demolition operations. The framework integrates four complementary measurement domains: environmental monitoring of crater soil, perimeter soil, water, surface dust, fragments, debris and equipment contamination; personal exposure monitoring using inhalable and respirable air sampling, real-time particulate monitoring and surface-wipe assessment; short-term urinary monitoring versus periodic or longitudinal blood sampling; and contextual and operational data covering task duration, entry frequency, protective equipment, decontamination procedures, meteorology and demolition characteristics. Field blanks, duplicates, chain-of-custody procedures and laboratory quality assurance and quality control are incorporated to support data integrity. Together, these sampling streams are intended to identify when and where exposure occurs, distinguish inhalation, dermal and secondary-transfer pathways, evaluate accumulation across repeated cycles, assess control effectiveness and support exposure reconstruction and epidemiological investigation. The figure is conceptual and does not represent measurements from a specific operation or an estimate of personal dose. PPE = personal protective equipment. QA/QC = quality assurance/quality control.

Validation of the proposed exposure pathway will require measurements aligned with the full operational sequence rather than isolated post-demolition soil sampling. Environmental, personal, biological and contextual measurements should be collected from the pre-operation baseline through the detonation event and immediate plume, plume transport and deposition, crater re-entry, fragment recovery, preparation of subsequent demolition stacks, repeated demolition cycles and final decontamination. The proposed field-validation design is summarised in Figure 5 and Figure 6. This approach would permit separation of direct plume inhalation, later soil-disturbance exposure, direct surface transfer and secondary contamination, while also determining whether residues and worker exposure accumulate across repeated demolition cycles.

The design integrates four complementary sampling streams: environmental monitoring of soil, fragments, dust and surfaces; personal monitoring of inhalable and respirable particulate exposure; biological monitoring for DNT metabolites or other validated biomarkers; and detailed contextual records covering task duration, weather, demolition characteristics, protective equipment and decontamination procedures. Together, these data would allow the screening model to be replaced by task-specific estimates of breathing-zone concentration, dermal loading, transfer efficiency and cumulative exposure.

## 4. Conclusions

This study reframes repeated mixed-munition demolition as a source-pathway-receptor problem in which DNT-bearing material may enter a primary post-blast plume, deposit within and beyond a demolition pit, and be resuspended or transferred during subsequent operational tasks. DNT is a toxicologically relevant candidate because it may occur as a TNT manufacturing impurity, as a constituent of other energetic materials and as part of mixed nitroaromatic residue profiles. The engineering characteristics of heterogeneous donor-initiated stacks support the plausibility of incomplete reactions, direct airborne release and residue generation.

Under the specified screening assumptions, the potential post-reaction DNT mass before application of any local-retention factor ranged from 5.00 to 200 g. Conditional locally retained mass ranged from 2.50 to 100 g at 50% retention, 3.75 to 150 g at 75% retention, and 4.50 to 180 g at 90% retention. These values do not constitute measurements of the Operation LEATHERMAN crater and should not be interpreted as a reconstruction of the original chemical inventory. The 1000 kg source basis is the documented aggregate TNT-equivalent operational value; it was neither calculated as 20 × 50 kg NEQ = 1000 kg TNT equivalent nor analytically verified as a mass of chemical TNT. The itemised inventory indicates that approximately 90% of the aggregate energetic equivalence was contributed by TNT-filled munitions. Scaling the documented basis by this contribution gives an approximately 900 kg first-order comparator; substituting that comparator would reduce every modelled DNT mass by 10%, including the potential post-reaction range to 4.50 to 180 g, without altering the qualitative interpretation. Retaining 1000 kg is therefore mildly conservative relative to this comparator, while the larger uncertainties remain the exact fill masses, TNT purity, DNT concentration, residual energetic-material fraction, and local retention. The illustrative retention fractions are sensitivity points rather than measured DNT-specific deposition or fallback factors. A non-zero conditional local DNT burden is obtained under the lowest-residue, lowest-DNT, and 50% retention scenario, although this does not establish actual retention, mobilisation, or worker exposure. Fine soil and contaminated particulate matter may nevertheless have been transported beyond the visible crater area, and the actual local burden cannot be determined retrospectively.

No comparison with an occupational exposure limit can be made from the scenario-estimated residue masses or crater dimensions. Such a comparison requires time-resolved monitoring of the immediate post-blast plume and task-specific personal breathing-zone measurements during re-entry, supported by particle-size-resolved sampling, chemical analysis of collected particulate matter, vapour-phase sampling where feasible, meteorological observations, exposure-duration data and records of respiratory and dermal protection use. Dermal loading and incidental ingestion require separate assessment because airborne concentration limits do not represent total systemic uptake.

Despite these limitations, the model identifies a coherent and biologically plausible pathway that warrants direct investigation. The proposed urothelial mechanism links metabolic activation and urinary delivery of DNT-derived species with oxidative or electrophilic DNA damage and imperfect repair, but each transition remains to be demonstrated directly in human urothelial models. The most appropriate next step is not to infer dose or disease risk from retained mass, but to undertake integrated plume characterisation, environmental sampling, personal monitoring, surface assessment and biological monitoring during representative demolition operations. These data would enable refinement of source inventory, direct atmospheric release, deposition, secondary mobilisation, task-specific exposure and cumulative-dose components of the model.

The model does not establish that DNT caused the reported excess of bladder cancer among former ammunition technicians. However, it provides a testable hypothesis and a structured basis for determining whether DNT and other demolition-related contaminants contribute materially to the occupational exposome of ammunition technicians and explosive ordnance disposal personnel.

## Figures and Tables

**Table 1:** Principal assumptions and their implications for interpretation.

Assumption or Data Gap	Effect on the Model	Required Evidence for Refinement
Documented 1000 kg TNT-equivalent operational basis used as a screening proxy for the TNT-bearing source	The 1000 kg value is an aggregate operational TNT-equivalent basis, not a measured chemical TNT mass and not a value derived from 20 × 50 kg NEQ. Approximately 90% of the aggregate energetic equivalence was contributed by TNT-filled munitions. A 900 kg proportional comparator therefore provides a source-basis sensitivity check; using it would reduce every DNT mass output by 10% without changing the model interpretation.	Item-specific explosive fill masses, formulation records, TNT purity data, and chemical analysis of DNT content
Residual energetic fraction applied across the entire mixed stockpile	Does not distinguish high-order, partial and low-order outcomes by munition type	Post-event residue recovery and formulation-specific detonation-efficiency data
DNT fraction applied uniformly to residual material	Assumes similar DNT content across diverse fills and items	Chemical analysis of source fills or composition-specific scenario ranges
DNT survival follows the residual-material scenario	Does not account for DNT-specific destruction, volatilisation or transformation	Post-detonation DNT mass balance and speciation
Illustrative local-retention fractions of 50%, 75% and 90%	Sensitivity values are not DNT-specific measured deposition or fallback fractions and do not represent lower, central or upper estimates of the true value	Spatially resolved sampling of the crater floor, walls, rim, immediate margins and material transported beyond the local working area
Qualitative inference from operational photographs and pit configuration	Coarse ejecta may be visible, but fine-particle transport cannot be quantified; repeated pit reworking and donor-charge placement do not determine a numerical retention fraction	Contemporaneous particle-size-resolved mass balance and spatial sampling during representative demolition operations
Residue considered available for mobilisation	Does not quantify dilution through soil, hotspots, sorption, ageing or bioavailability	Soil concentration, particle-size and desorption/bioavailability measurements
Dimensional mass-volume illustration	Artificial uniform dispersion through the crater volume does not represent an open atmosphere, task-generated aerosol or personal breathing-zone exposure	Task-resolved personal air sampling, chemical analysis of collected dust, meteorology and exposure-duration data
No exposure duration or frequency term	Cannot calculate an 8 h TWA or cumulative dose	Task timing, entry frequency and longitudinal work-history data
Dermal and ingestion pathways described qualitatively	Cannot estimate route-specific absorbed dose	Surface loading, glove/skin wipes, hand-to-mouth observations and biomonitoring
Disease association not modelled	Cannot attribute bladder cancer or other outcomes to DNT	Exposure-reconstructed epidemiology with confounder and co-exposure control
Illustrative conversion of crater volume to equivalent soil mass using 1350 kg/m^3^	The apparent crater is a geometric void and the bulk density was not measured; the calculation does not estimate the actual mass or spatial distribution of contaminated soil	Site-specific bulk-density measurements, three-dimensional reconstruction of disturbed soil and spatially resolved DNT analysis
Primary blast-generated DNT aerosol and vapour not quantified	The model estimates potential and conditionally retained mass but does not estimate direct airborne release, vapour-particle partitioning, plume concentration, atmospheric residence or downwind transport.	Time-resolved post-blast air sampling with particle-size-resolved filters, real-time aerosol monitoring, vapour-phase collection where feasible, meteorological measurements and plume-dispersion reconstruction.

**Table 2:** Scenario inputs used in the screening model.

Parameter	Scenario Value	Interpretive Status
Aggregate TNT-equivalent basis	1000 kg	Documented aggregate energetic-equivalence basis for the operation; not derived as 20 × 50 kg NEQ. Approximately 90% of the aggregate equivalence was contributed by TNT-filled munitions. Used as a mildly conservative screening proxy, not an analytically verified chemical TNT mass.
Number of demolition events	20	Sequential demolitions in one pit
Nominal NEQ per event	Approximately 50 kg NEQ	Operationally documented stack limit; reported as NEQ and not converted to TNT equivalence
Gross recovered ammunition and explosives	Approximately 60 tonnes	Gross operational stockpile mass, including munition bodies and other non-energetic material; contextual only and not used as a model explosive-mass input.
TNT-filled share of aggregate energetic equivalence	Approximately 90%	Indicated by the itemised operational inventory; the principal non-TNT component comprised predominantly RDX-filled PG-7 variants.
Proportional TNT-filled source-basis comparator	Approximately 900 kg	Calculated as 90% of the documented 1000 kg aggregate basis for sensitivity only. It is not an independent reconstruction of chemical TNT mass; substituting it would reduce every modelled DNT mass by 10%.
Residual energetic material fraction	0.5%, 1.0% and 2.0%	Screening range selected to represent limited residual energetic material without implying widespread inefficient functioning
DNT fraction applied to residual material	0.1%, 0.5% and 1.0%	Scenario range for uncertain legacy-material composition
Illustrative local-retention fractions	50%, 75% and 90%	Sensitivity points only; not measured fallback or deposition rates and not lower, central or upper estimates of the true value
Crater geometry	Semi-ellipsoid	Based on measured final dimensions
Semi-major axis	4.0 m	Half of the measured 8.0 m crater length
Semi-minor axis	3.5 m	Half of the measured 7.0 m crater width
Depth	1.9 m	Measured final crater depth

**Table 3:** Potential post-reaction DNT mass and conditional locally retained mass across illustrative local-retention fractions.

Residual Energetic Material Fraction (Mass)	Applied DNT Fraction	Potential Post-Reaction DNT Mass *	Locally Retained DNT at 50%	Locally Retained DNT at 75%	Locally Retained DNT at 90%
0.5% (5 kg)	0.1%	5.00 g	2.50 g	3.75 g	4.50 g
0.5% (5 kg)	0.5%	25.0 g	12.5 g	18.8 g	22.5 g
0.5% (5 kg)	1.0%	50.0 g	25.0 g	37.5 g	45.0 g
1.0% (10 kg)	0.1%	10.0 g	5.00 g	7.50 g	9.00 g
1.0% (10 kg)	0.5%	50.0 g	25.0 g	37.5 g	45.0 g
1.0% (10 kg)	1.0%	100 g	50.0 g	75.0 g	90.0 g
2.0% (20 kg)	0.1%	20.0 g	10.0 g	15.0 g	18.0 g
2.0% (20 kg)	0.5%	100 g	50.0 g	75.0 g	90.0 g
2.0% (20 kg)	1.0%	200 g	100 g	150 g	180 g

* Potential post-reaction DNT mass was calculated before applying any local-retention factor. Conditional locally retained masses were calculated using illustrative retention fractions of 50%, 75% and 90%. These values are sensitivity points selected to test the robustness of the proposed accumulation pathway; they are not measured fallback or deposition rates and are not presented as lower, central and upper estimates of the true value. The local environment includes the crater floor, walls, rim and immediately adjacent disturbed soil. The calculation estimates the fraction of the residual DNT burden remaining locally and does not assume that DNT and soil behaved identically during detonation. Values are rounded to three significant figures. A 900 kg proportional source-basis comparator (90% of the documented aggregate basis) would multiply every value in this table by 0.90; for example, the potential post-reaction range would be 4.50 to 180 g. This comparator is not an independent reconstruction of chemical TNT mass.

## Data Availability

All model data is presented in the tables.

## Abbreviations

The following abbreviations are used in this manuscript:

DNT	Dinitrotoluene
TNT	Trinitrotoluene
